# Toward Isolation of Palytoxins: Liquid Chromatography Coupled to Low- or High-Resolution Mass Spectrometry for the Study on the Impact of Drying Techniques, Solvents and Materials

**DOI:** 10.3390/toxins13090650

**Published:** 2021-09-14

**Authors:** Antonia Mazzeo, Michela Varra, Luciana Tartaglione, Patrizia Ciminiello, Zita Zendong, Philipp Hess, Carmela Dell’Aversano

**Affiliations:** 1 Department of Pharmacy, School of Medicine and Surgery, University of Napoli Federico II, Via D. Montesano 49, 80131 Napoli, Italy; antonia.mazzeo@unina.it (A.M.); varra@unina.it (M.V.); ciminiel@gmail.com (P.C.); dellaver@unina.it (C.D.); 2 CoNISMa—National Inter-University Consortium for Marine Sciences, Piazzale Flaminio 9, 00196 Rome, Italy; 3 Ifremer, DYNECO, Laboratoire Phycotoxines, Rue de l’Île d’Yeu, 44311 Nantes, France; Zita.Zendong@effem.com (Z.Z.); Philipp.Hess@ifremer.fr (P.H.)

**Keywords:** palytoxin, LC-HRMS, evaporation, stability, palytoxin methyl ester

## Abstract

Palytoxin (PLTX) and its congeners are emerging toxins held responsible for a number of human poisonings following the inhalation of toxic aerosols, skin contact, or the ingestion of contaminated seafood. Despite the strong structural analogies, the relative toxic potencies of PLTX congeners are quite different, making it necessary to isolate them individually in sufficient amounts for toxicological and analytical purposes. Previous studies showed poor PLTX recoveries with a dramatic decrease in PLTX yield throughout each purification step. In view of a large-scale preparative work aimed at the preparation of PLTX reference material, we have investigated evaporation as a critical—although unavoidable—step that heavily affects overall recoveries. The experiments were carried out in two laboratories using different liquid chromatography-mass spectrometry (LC-MS) instruments, with either unit or high resolution. Palytoxin behaved differently when concentrated to a minimum volume rather than when evaporated to complete dryness. The recoveries strongly depended on the solubility as well as on the material of the used container. The LC-MS analyses of PLTX dissolved in aqueous organic blends proved to give a peak intensity higher then when dissolved in pure water. After drying, the PLTX adsorption appeared stronger on glass surfaces than on plastic materials. However, both the solvents used to dilute PLTX and that used for re-dissolution had an important role. A quantitative recovery (97%) was achieved when completely drying 80% aqueous EtOH solutions of PLTX under N_2_-stream in Teflon. The stability of PLTX in acids was also investigated. Although PLTX was quite stable in 0.2% acetic acid solutions, upon exposure to stronger acids (pH < 2.66), degradation products were observed, among which a PLTX methyl-ester was identified.

## 1. Introduction

Palytoxin (PLTX) is a very potent marine toxin contained in several species of marine zoanthids belonging to the genus *Palythoa* [1,2] as well as in cyanobacterial blooms *Trichodesmium* sp. [3]. A number of PLTX analogues have been detected in sub-tropical microalgae belonging to the genus *Ostreopsis* and named ovatoxins from *O.* cf. *ovata* and *Ostreopsis fattorussoi*, ostreocins from *O. siamensis* and mascarenotoxins from *O. mascarenensis* [4,5,6]. Palytoxin and its congeners are large molecules of polyketide nature with a molecular weight > 2600 Da, featuring a polyhydroxylated and a partially unsaturated aliphatic chain containing assorted cyclic ether, amine, amide and ketal/hemiketal functionalities (Figure S1).

Although the first cases of poisonings referred to the ingestion of PLTX contaminated seafood [7], several cases of respiratory poisonings and/or skin injuries have been reported in beachgoers during massive blooms of *O.* cf. *ovata* [8] as well as in aquarium hobbyists from incidental contact with PLTX-producing *Palythoa* spp. [9,10]. The symptom similarities between the *Ostreopsis-* and *Palythoa-*related poisonings suggested that PLTXs are the etiological agents and the respiratory toxicity of PLTXs was successively proven by the exposure of rats to aerosolized PLTX preparations [9,11]. Nevertheless, the toxicity studies performed on a few PLTX congeners showed that, despite small diversity in structure or even in stereo-structure, their relative toxic potencies might be quite different either *in vivo* or *in vitro* [10,12,13,14,15,16]. As a consequence, the need exists to evaluate the individual toxicity of each PLTX congener in order to carry out reliable structure–activity relationship studies and assess the real hazard they present to humans. The availability of sufficient amounts of well characterized reference material is an important pre-requisite to obtain toxicity data. To this aim, an isolation procedure for the quantitative recovery of each PLTX congener should be developed and the stability of PLTX under different conditions should be evaluated.

Previous work by Ciminiello et al. on the isolation of ovatoxin-a, the main PLTX-like toxic component of *O.* cf. *ovata* [17], although successful in recovering 700 µg of pure toxin from 80 liters of algal culture, only provided an overall recovery of about 12% as the result of extraction, four chromatographic and multiple evaporation steps. Similarly, Brissard et al. [18] obtained an 85% recovery of ovatoxins after a single chromatography; however, the same authors only recovered a third of the expected toxins after complete evaporation. In the evaluation of polymeric sorbents for the passive sampling of marine toxins from seawater, Zendong et al. also reported poor recoveries for ovatoxin-a [19]. Even the isolation of assorted PLTXs from *P. tuberculosa* required 45 kg of starting material to end up with only 4 mg of pure individual toxins [2]. There is no experimental evidence to explain whether the low recoveries of PLTX and its congeners are due to the instability of the compounds in solution or to the irreversible adsorption to materials or even to other uncontrolled factors. Only Taylor et al. suggested a non-specific binding of PLTX to polyethylene tubing [20]. 

In view of large-scale isolation work, the causes underlying the significant losses in PLTX need to be clarified. Since the evaporation step is unavoidable in any isolation protocol, in this study, we investigated the recoveries of PLTX in different samples under various experimental conditions including the use of different evaporation systems, as well as complete drying versus concentration to a minimum volume. The influence on the toxin recovery of various solvents (aqueous or organic) and of the most common container materials (different qualities of glass and plastics) was investigated. Based on the possibility to add acids in mobile phases to be used in some of the chromatographic steps, the stability of PLTX under various acidic conditions was also investigated. The experiments were carried out in two laboratories using different LC-MS instruments operating at unit and high resolution. For the sake of clarity, a schematic summary of the overall experiments has been reported in Table 1.

## 2. Results and Discussion

### 2.1. Preliminary Evaporation Experiment of OA and PLTX

In order to substantiate the heavy toxin losses reported within the purification of PLTXs [17,18,19] and to exclude that they were due to an operators’ systematic bias, a preliminary evaporation experiment was performed on a mixture of PLTX and okadaic acid (OA), a diarrhetic shellfish poisoning toxin that was used as reference to compare drying down recoveries. An equimolar solution of PLTX and OA in 80% aqueous MeOH—the solvent most employed to extract PLTXs from mussels [21,22] and seawater [9]—was evaporated to dryness under N_2_-stream. A statistically significant lower recovery was measured for PLTX (62 ± 6%; *n* = 3) than that obtained for OA (103 ± 2%; *n* = 3). This clearly confirmed the previously reported data [17,18,19] and suggested that at least one of the reasons for toxin loss is related to the drying down step, which, therefore, represents a key step to optimize within the isolation work.

### 2.2. Effect of Solvents on the Intensity of the [M+H+Ca]^3+^ Ion of PLTX

**Figure 1:**
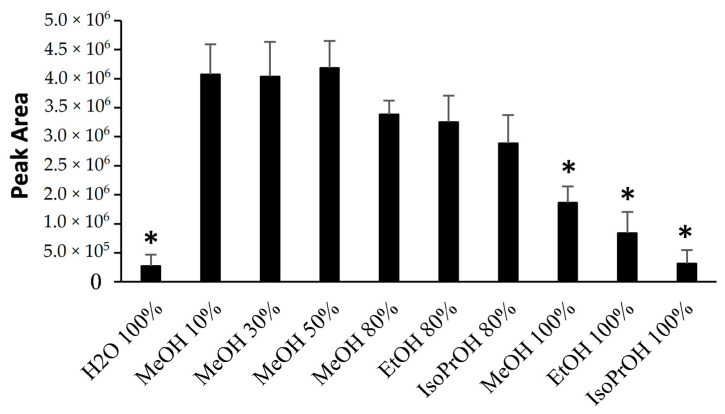
LC-HRMS response (peak areas of the Extracted Ion Chromatogram at *m/z* 906.4824) of PLTX (1.0 µg/mL) stored in various solvents mixtures in glass vials. Error bars represent standard deviation (*n* = 3). Response in 50% aqueous MeOH was taken as reference. * denotes statistically significant difference, *p* < 0.001.

The [M+H+Ca]^3+^ ion is the base peak in the full scan HRMS spectrum of PLTX and its congeners analyzed under the conditions described above, and it is the main ion used for quantitative purposes. In order to identify the effect of the solvent condition on the intensity of the [M+H+Ca]^3+^ ion, PLTX stock solution was diluted (1:10) in different solvent mixtures (Table 1) in glass vials and analyzed using LC-HRMS 2h after preparation (Figure 1 and Table S2).

The highest responses were obtained when PLTX was diluted in 10%, 30% and 50% aqueous MeOH with no significant difference among the three blends. A decreasing trend in the response was observed for the 80% aqueous blends, in the order 80% MeOH > 80% EtOH > 80% IsoPrOH. Taking the PLTX response in 50% aqueous MeOH as a reference (control), a significantly lower response (*p* < 0.001) was observed for PLTX dilutions in pure organic solvents (MeOH > EtOH > IsoPrOH). A 93% decrease in the PLTX response versus the control was also recorded for PLTX in water, suggesting that an appropriate amount of organic modifier appears suitable for the detection of PLTX using LC-HRMS. 

**Figure 2:**
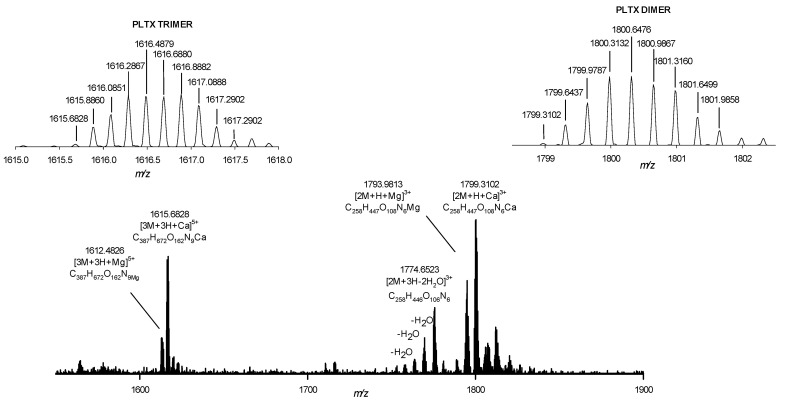
Full HRMS spectrum of PLTX (*m/z* 1510–1900) where PLTX dimer and trimer ion clusters appear dominated by [2M+H+Ca]^3+^ and [3M+3H+Ca]^5+^ ions, respectively. Formula assigned to monoisotopic ion peaks are reported together with the isotopic ion patterns of the most intense ions.

The low PLTX response in water could be due to the formation of ion species different from those monitored in the LC-HRMS experiments, such as a PLTX dimer [23]. To test this hypothesis, the full HRMS spectra of PLTX standard (10.0 µg/mL) in pure water (Figure S2) and in 50% aqueous MeOH were acquired (Figure 2). In both samples, the presence of a PLTX dimer [2M+H+Ca]^3+^ at *m/z* 1799.3102 (Δ = 0.479 ppm) and a PLTX trimer [3M+3H+Ca]^5+^ at *m/z* 1615.6828 (Δ = −0.997 ppm) emerged (Figure 2), together with the monomer [M+H+Ca]^3+^ and [M+2H-H_2_O]^2+^ at *m/z* 906.4824 and *m/z* 1331.2412, respectively. The PLTX monomer represented the dominant form in the spectra of both samples, with the dimer accounting only for 2.6% (in water) and 1.6% (in 50% aqueous MeOH) of the total ion species contained in the spectra. Even when assuming that the dimer and trimer have reduced ionization yield, this suggested that the ionization behavior of PLTX in the two solutions was similar and that the dimer/trimer-formation did not significantly affect the reduction in response. Overall, these observations suggested that another issue deserves attention to interpret the low LC-HRMS response of PLTX in water and we focused on the hypothesis of the adsorption of PLTX on the container surfaces [20].

Previous studies on the adsorption of organic molecules on a silica surface revealed that the hydration forces, originated from the water structure in the vicinity of silica, are disrupted in the presence of methanol. Methanol adsorbs on silica by the displacement of water molecules [24,25]. Furthermore, ethanol also adsorbs on silica to a lesser extent than methanol. This phenomenon could account for our experimental data suggesting that the PLTX adsorption occurs by an interaction between PLTX itself, or its water solvated form, with hydrated glass. The 10% organic component in methanol aqueous blends, disrupting the water–glass layer, inhibits PLTX adsorption. Therefore, the intensity of the [M+H+Ca]^3+^ ion of PLTX, strongly increases (up to 13-fold) in water-based organic blends compared to PLTX in 100% water (Table S2). The increase in the percentage of MeOH in aqueous blends from 10 to 50% (Figure 1) did not result in a further increase in the intensity of the PLTX LC-HRMS signal. If we also include the percentage of methanol contained in the PLTX stock solution, the PLTX sample labelled as 10% methanol in water was actually 15% methanol in water and the obtained results are in full agreement with previous data that show that about 15% MeOH in water is sufficient to disrupt the water–silica layer [24,25,26]. 

The behavior of PLTX in water shows similarity to that of microparticles on solid surface where the liquid meniscus formed around the contact area between the microparticle and the surface, due to the condensation of water, results in an additional capillary force between the two materials [26]. 

Moreover, the relative intensity of the PLTX [M+H+Ca]^3+^ ion in organic-aqueous mixtures containing more than 50% organic solvent as well as in pure organic solvent decreased according to the dielectric constant of the organic component (MeOH>EtOH>IsoPrOH), thus suggesting that an aqueous mixture of an opportune organic solvent promotes its dissolution. 

In conclusion, handling PLTX requires that even a simple dilution pre-treatment should be carefully performed and that, despite the high polarity of the toxin, the low response (Figure 1) in 100% water using glass vials leads to consider this condition inappropriate.

### 2.3. Effect of Drying Techniques on PLTX Recovery

**Figure 3:**
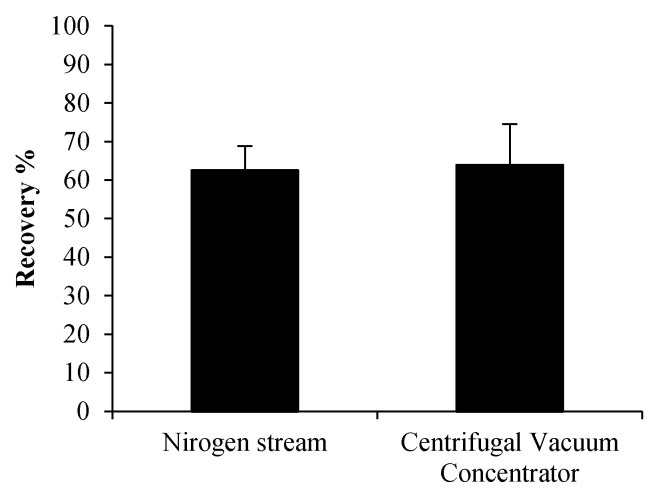
Palytoxin Recoveries after complete drying under Nitrogen stream and Centrifugal Vacuum Concentrator (see Table 1 for experimental details).

PLTX solutions (Table 1, condition 3, 80% aqueous MeOH) were dried down using N_2_-stream or a Centrifugal Vacuum Concentrator in glass vials without a cap (as routinely conducted) over one day. Based on the results reported in Figure 1, after drying down, each PLTX residue was re-dissolved using 50% aqueous MeOH. The LC-MS analyses (Figure 3) showed that the PLTX recoveries were quite similar using N_2_-stream (62 ± 6%) and a Centrifugal Vacuum Concentrator (64 ± 5%). This suggested that the PLTX recovery is affected to the same extent by both evaporation techniques. Due to the way in which the two techniques are conceived, it cannot be excluded that within the dry-down processes, the above-described strong solvent-mediated adsorption forces trigger incipient motions from which a partial loss of PLTX could be favored [8,9,10,26].

However, the recovery being unsatisfactory for multi-step purification from natural matrices, we then explored the drying down using different materials for tubes and/or vials.

### 2.4. Complete Drying in Different Materials

**Figure 4:**
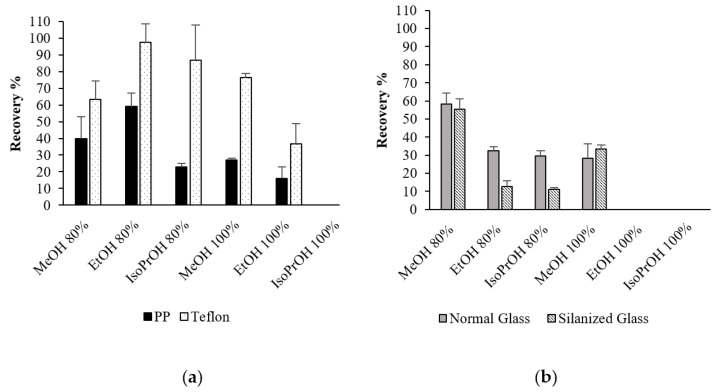
Recoveries of PLTX (125 ng/mL, 1.0 mL) following complete drying performed with N2-stream in (**a**) PP and Teflon tubes and (**b**) normal and silanized glass vials. The residues were re-dissolved in 300 μL of the initial solvent/blend. Recoveries are expressed in % ± RSD as mean of three different replicates.

The interaction between the tube materials and the PLTX dissolved in pure MeOH, EtOH and IsoPrOH, as well as 80% aqueous blends of each of the above solvents, was investigated, performing complete drying experiments under N_2_-stream in normal and silanized glass vials as well as in PP and Teflon tubes (Figure 4a,b and Table 1 and Table S3). The highest recoveries were obtained when PLTX was dried down in Teflon tubes, with a 97% recovery obtained for the 80% aqueous EtOH sample. In both plastic materials, the recoveries in pure organic solvents (MeOH > EtOH >> IsoPrOH) paralleled the trend of the data described in Section 2.2 (Figure 1 and Figure 4a). In both glass materials (Figure 4b), unsatisfactory recoveries were obtained for all the tested solvents. PLTX was not detected after drying down in pure EtOH and IsoPrOH, with only 33% being recovered after drying down in MeOH. Even considering the good solubility of PLTX in aqueous blends, no more than 58% could be recovered after drying it down from aqueous mixtures, thus supporting the hypothesis above-described in Section 2.2.

The PLTX recovery in PP tubes and silanized glass vials appears similar to that obtained diluting PLTX in different solvents or blends (see Section 2.2). To test this hypothesis, we also performed the complete drying of a new set of PLTX samples in PP tubes using MeOH, EtOH and IsoPrOH and 80% aqueous blends of each of such solvents as starting solvents and re-dissolving all the samples in 50% aqueous MeOH. The results are shown in (Table 2). As expected, higher recoveries were obtained for all the samples when re-dissolved in 50% MeOH. However, overall recoveries were still unsatisfactory (40–61%), thus suggesting that adsorption could also occur on the surface of these two types of materials, mainly through Van der Waals interactions.

**Figure 5:**
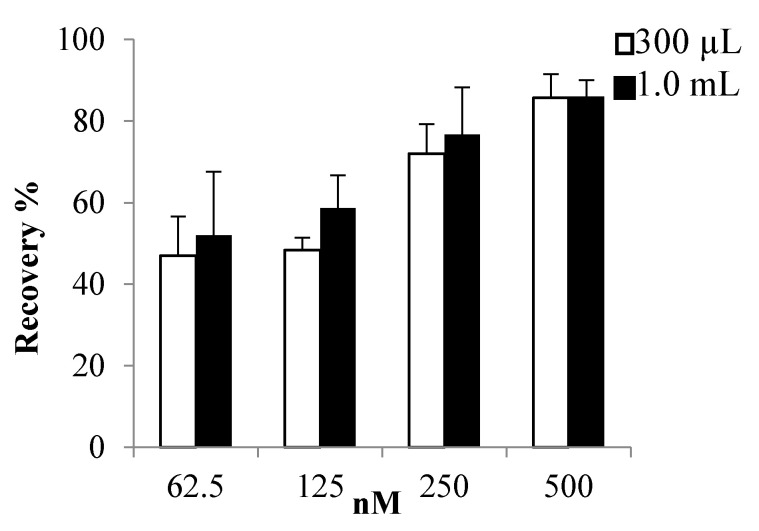
Recoveries of PLTX at different initial concentration (initial volume 1.0 mL, in 80% aqueous MeOH) following complete drying performed with N_2-_stream in Teflon tubes. The samples were re-dissolved in 300 µL or 1.0 mL of 50% aqueous MeOH. Recoveries are expressed in % ± RSD as mean of three different replicates.

Due to the positive results obtained in Teflon tubes, and considering the different results obtained for 80% aqueous MeOH or EtOH blends (Figure 4a), we also explored PLTX recoveries following drying down under N_2_-stream in Teflon tubes using different concentration levels (62.5, 125, 250 and 500 ng/mL) in 80% aqueous MeOH (Figure 5 and Table S4). The residues were re-dissolved, either in 300 µL or in 1 mL of 50% aqueous MeOH. The recoveries increased with the concentration, with the highest recovery for PLTX (86%) being obtained at the highest concentration and the lowest recoveries (about 47%) at the lowest concentrations (<250 ng/mL).

Increasing the re-dissolution volume from 300 µL to 1.0 mL, recoveries improved by 5–11% at the lowest concentration levels (62.5 to 250 ng/mL), while no difference was observed at 500 ng/mL.

Despite the fact that the adsorption forces are strongly reduced on Teflon tubes, some PLTX loss through incipient motions could occur according to the used drying technique. From the data reported in Table S4, it emerges that PLTX loss reaches its maximum of 60–70 ng, already at a PLTX concentration of 125 ng/mL. However, at the lowest explored PLTX concentration (62.5 ng/mL), only 30 ng of PLTX were lost. 

### 2.5. Concentration in Different Materials

**Figure 6:**
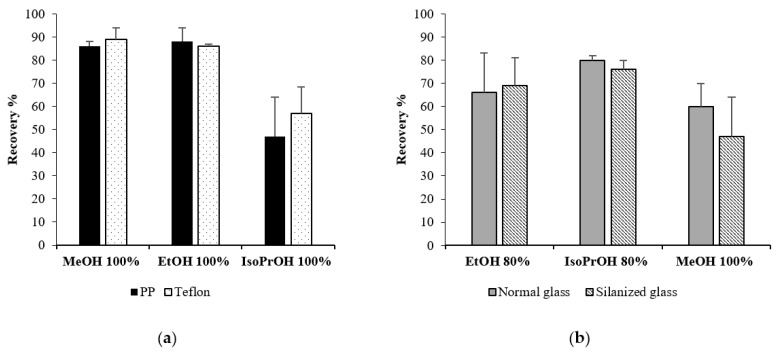
Recoveries of PLTX (125 ng/mL, 1.0 mL) following concentration to 200 µL under N_2_-stream in (**a**) PP and Teflon tubes and (**b**) normal and silanized glass vials. Recoveries are expressed in % ± RSD as mean of three different replicates.

In order to further explore the phenomena occurring during drying down, we chose to stop some of the above described experiments that gave the worst results (Figure 4) to the intermediate step of concentration (not evaporating to dryness but concentrating to a smaller volume). Thus, the recovery after concentrating PLTX samples (125 ng, 1.0 mL) in 100% organic solvents to 200 µL under N_2_-stream was evaluated in PP and Teflon tubes (Figure 6a). Similarly, the recovery of PLTX, 125 ng in 1.0 mL of 80% EtOH or isoPrOH, was assessed after concentrated to 200 µL under N_2-_stream using normal and silanized glass vials (Figure 6b and Table S5). As expected, the fact that at the end of the process, PLTX was still kept in the solution, limited its adsorption on the surfaces (tubes/vials) that could catalyze the deleterious PLTX aggregation phenomena. In particular, we hypothesize that, since PLTX is a high molecular weight compound containing both polar and hydrophobic groups, its behavior in solution could be affected by the environmental conditions in a similar manner as that reported for proteins. Actually, proteins can aggregate into particles because of conformational changes caused by different mechanical and chemical–physical stress conditions (pH, temperature, pressure, vortexing, etc.) including surface adsorption, which, in turn, can promote aggregate formation [27,28]. This resulted in a higher recovery than that obtained after complete drying (Figure 4).

### 2.6. Study on Stability of PLTX in Acids

The extraction of PLTXs from algae and seawater is often performed using acidic conditions [9,29]. Furthermore, acids are frequently used in chromatography to improve the resolution of closely eluting analogs [30]. Therefore, the potential degradation of PLTX in acidic conditions was investigated. The tested acidic concentrations were those routinely used for PLTX purification [9,17,29]. Solutions of PLTX with additions of either acetic acid (AA) (pH 3.25), formic acid (FA) (pH 2.66), or trifluoro acetic acid (TFA) (pH 1.20) were analyzed at day 0 and at day 14 versus a control (PLTX in 80% aqueous MeOH). No statistically significant difference was measured at day 0 among the different treatments. At day 14, while no variations were observed for PLTX in AA versus control, a 64 and a 29% decrease in the PLTX signal was measured for the samples in TFA and FA, respectively. This suggested that exposure to strong acidic conditions (pH ≤ 2.66) causes PLTX degradation (Table 3).

**Figure 7:**
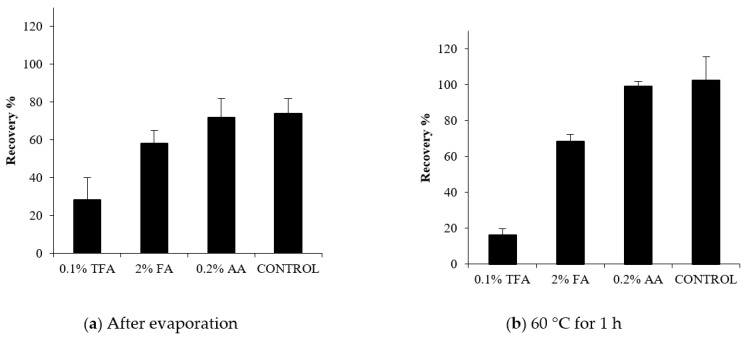
Recoveries of PLTX (125 ng) in 80% aqueous MeOH with the addition of 0.2% acetic acid (AA), 0.1% trifluoroacetic acid (TFA) and 2% formic acid (FA) and no acid (control) following (**a**) complete drying in Teflon tubes and subsequent re-dissolution in 1 mL of 80% aqueous MeOH and (**b**) heat-treatment (60 °C, 1.0 h) in glass tubes. Error bars represent standard deviation from mean of three different replicates.

The degradation in TFA and FA was even more evident following either complete drying under N_2_-stream (Figure 7a) or heat treatment (Figure 7b). The complete drying of PLTX in TFA and FA provided recoveries of 28 and 58%, respectively, likely as a consequence of a progressive decrease in the pH during the evaporation. Heat treatment (60 °C, 1 h) led to a dramatic loss of PLTX, with a recovery of 16% in TFA.

In both experiments, no significant difference occurred between PLTX in AA and in the control.

**Figure 8:**
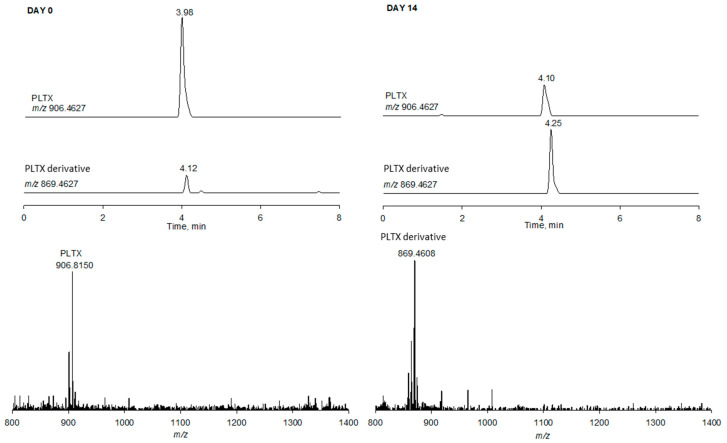
LC-HRMS analysis (flow rate 0.4 mL/min) of PLTX in 80% aqueous MeOH with 0.1% TFA (pH 1.20) at day 0 and 14. XICs of *m/z* 906.4627 (PLTX) and *m/z* 869.4627 (PLTX derivative) and full HRMS spectrum associated to each peak.

The PLTX degradation observed under the stronger acidic conditions can be associated with the presence of several acid-sensitive functionalities in PLTX structure (ketal, hemiketal, amide, enamide, hydroxyl groups). While the opening of the cyclic ketal/hemiketal functional group would not result in mass shifts of the ions in the full HRMS spectrum, hydrolysis of the amide/enamide or the dehydration of alcohols, or even the conversion of the hemiketal at C47 into a methyl ketal would lead to significant mass shifts. In order to investigate the structural changes occurring in PLTX under acidic conditions, we in-depth analyzed the full HRMS spectra of 0.1% TFA samples at day 0 and 14 (Figure 8 and Figure S3).

The presence of a triply charged ion at *m/z* 869.4608 (monoisotopic at *m/z* 869.1261, C_124_H_216_NO_53_Ca, RDB 17.5, Δ = −2.455 ppm) eluting in the vicinity of PLTX, emerged in all the acidified samples with the only exception of AA at day 0 and gradually increased overtime and with acid strength (Table 3), thus suggesting it was due to a degradation phenomenon. 

The difference of C_5_H_8_N_2_O between the molecular formulae of the PLTX derivative (C_124_H_215_NO_53_) and PLTX (C_129_H_223_N_3_O_54_) suggested that the structural modification occurs in the A-side terminal of PLTX where 2 N are present. It is most likely that such a mass difference could derive from either methanolysis of the amide at C1 (Figure S1), or the concomitant hydrolysis of such amide and the formation of a methyl ketal at C47, as outlined for the formation of azaspiracids artifacts [31]. The interpretation of the HRMS^2^ spectrum of the PLTX derivative according to Ciminiello et al. [32] allowed us to rule out the latter hypothesis. Indeed, the characteristic B-side fragments (cleavage #4, #5, #12, #13, #15, #17) and the internal fragments (#4 + 12, #4 + 15, #4 + 16) of the PLTX derivative (Table 4) were superimposable to those of PLTX [32], while all the relevant A-side fragments differed from PLTX for C_5_H_8_N_2_O. In addition, the characteristic A-side fragment at *m/z* 327 (cleavage #4) of most PLTX congeners [32,33,34,35] was totally lacking in the HRMS^2^ spectrum of the PLTX derivative. All of these data pointed toward the structural modifications being located in the C8-A-side terminal and, thus, clearly suggested that the PLTX derivative was the PLTX methyl ester at C1 (Table 4). Its formation, facilitated by strong acidic conditions, could be explained according to a tentative mechanism (Table 4) that starts with the protonation of the carbonyl oxygen at C1, leading to a fully conjugated N-acyliminium cation. In methanol, the presence of such a good leaving group would favor the formation of the methyl ester at C1. This reaction would occur in place of the reported N-vinyl acetamide hydrolysis that leads to amides and ketones [36].

## 3. Conclusions

In view of developing an isolation procedure for the quantitative purification of individual PLTX congeners aimed at the preparation of RMs for each individual analog, we have demonstrated that adsorption on the surface of tubes or vials is a critical issue that may heavily affect the overall recoveries. The phenomenon of adsorption differently affects PLTX recovery depending on the solubility in the solvent system used, with an aqueous organic blend being the best choice in most of the explored cases as compared to pure organic solvents or pure water. Our findings lead to the suspicion that adsorption forces could be mediated by water molecules. The adsorption appeared more pronounced on glass surfaces (either normal or silanized) than on plastic materials (PP or Teflon). The use of a higher percentage of organic component in the blends (>50%) strongly reduced the PLTX signals, according to the decreased dielectric constant of the organic component. As PLTX is an amphiphilic molecule, this phenomenon could be related to the decrease in the PLTX solubility in the pure organic modifiers. To confirm this hypothesis, further investigations would be needed to explore the role of solubility in the PLTX losses emerged in this study.

PLTX behaved differently during concentration to a minimum volume than when completely dried down. The recoveries strongly depended on the solvent mixture used to re-dissolve the toxin as well as on the materials in which the drying down was carried out. Glass materials could be used only to concentrate aqueous EtOH and IsoPrOH solutions. When PLTX is dissolved in 100% MeOH, complete drying should be avoided and concentration is preferable, either in glass or plastic materials. In general, Teflon provided the best results, both when completely drying aqueous solutions and concentrating organic solutions to a minimum volume. The complete drying of 80% ethanolic solutions of PLTX in Teflon under Nitrogen stream provided a quantitative recovery (97%). 

The use of acids for the extraction or the chromatographic separation should be carefully considered. Although PLTX is quite stable in 0.2% AA solutions at room temperature, in good agreement with some isolation protocols [29], PLTX is not stable in stronger acidic conditions (pH < 2.66) generating degradation products. Among them, the PLTX methyl ester at C1 was identified.

## 4. Materials and Methods 

Statement of human and animal rights: This paper does not raise any concern regarding human and animal rights.

### 4.1. Chemicals and Standards

Organic solvents (HPLC grade) were from AtlanticLabo (Bordeaux, France) and Sigma Aldrich (Steinheim, Germany). Laboratory grade trifluoroacetic acid (TFA), acetic acid (AA), formic acid (FA) and water (HPLC grade) were from Sigma Aldrich (Darmstadt, Germany). Milli-Q water was produced in-house to 18 MΩ/cm quality, using a Milli-Q integral 3 system from Merck Millipore KGaA (Darmstadt, Germany). Normal glass vials (1.5 mL) were from Agilent (Santa Clara, CA, USA). Silanized glass vials (1.5 mL) were from Macherey-Nagel GmbH & Co. KG (Duren, Germany), Polypropylene (PP) and Teflon tubes (1.5 mL) were from Merck Millipore KGaA (Darmstadt, Germany). Certified standard of okadaic acid (OA) from National Research Council Canada (Halifax, NS, Canada) was used in preliminary experiments. A non-certified PLTX standard (100 μg; lot LAM7122) from Wako Chemicals GmbH (Neuss, Germany) was dissolved in 10 mL of 50% aqueous MeOH and used as stock solution (10 µg/mL) in all the experiments. We previously verified [8] that the authentic composition of this standard was 84% PLTX itself, 1.7% 42-hydroxypalytoxin and 14.3% other PLTX-like contaminants. Even so, in our calculations, we referred to the standard as consisting of 100% PLTX. Before each set of experiments described below, a seven-level PLTX calibration curve in 50% aqueous MeOH (1000, 500, 250, 125, 62.5, 31.2, 15.6 ng/mL) was used for quantitation. 

### 4.2. Detection Methods

Liquid chromatography-mass spectrometry at unit and high resolution (LC-MS and LC-HRMS) 

LC-MS experiments were carried out using a 2.7-micrometer Poroshell 120 EC-C18, 2.1 × 100 mm (Agilent, La Jolla, CA, USA) kept at 25 °C. Mobile phase was water (eluent A) and 95% acetonitrile/water (eluent B), both containing 30 mM AA. Gradient elution at flow rate of 0.2 mL/min was 20 to 100% B in 10 min, hold for 5 min before dropping down to the initial conditions with a re-equilibration time of 10 min. The analyses were carried out on the following two different instruments in triplicate:

Instrument 1 (Ifremer, France): An UFLC-XR Shimadzu LC system (Shimadzu, Japan) was coupled to a hybrid triple quadrupole/ion-trap mass spectrometer API 4000 Qtrap MS (ABSCIEX, Concord, ON, Canada) equipped with a TurboIonSpray^TM^ ionization source. Source settings were curtain gas, 30 psi; ion spray, 5000 V; turbogas temperature, 300 °C; declustering potential, 56 V; entrance potential, 10 V; Gas 1 and Gas 2, 30 and 40 psi, respectively. MS detection of PLTX and OA was carried out in multiple reactions monitoring (MRM) mode (positive ions, Dwell time 500 or 250 ms, for PLTX or OA, respectively) according to Table S1 and Figure S1. The sum of MRM peak areas was used to express peak intensity. Limit of Detection (LOD) was 15 ng/mL. Analyst 1.5 software was used for integration of peaks and quantitation.

Instrument 2 (University of Napoli Federico II, Naples, Italy): An Ultimate 3000 quaternary LC system was coupled to a hybrid linear ion trap LTQ Orbitrap XL FTMS equipped with an ESI ION MAX^TM^ source (Thermo-Fisher, Waltham, MA, USA). Full HRMS experiments for PLTX (positive ions) were acquired in the range *m/z* 800–1400 and/or *m/z* 1400–2000 at a resolving power (RP) of 60,000 (FWHM at *m/z* 400). Source settings were spray voltage, 4.8 kV; capillary temperature, 360 °C; capillary voltage, 17 V; tube lens voltage, 145 V; sheath gas and auxiliary gas flow, 32 and 4 (arbitrary units), respectively. HR collision induced dissociation (CID) MS^2^ experiments were acquired at a RP 60,000 using collision energy, 35%; isolation width, 3.0 Da; activation Q, 0.250; and activation time, 30 msec. Precursor ions were [M+H+Ca]^3+^ of PLTX (*m/z* 906.8) and PLTX methyl ester (*m/z* 869.4). Xcalibur software v2.0.7. was used to calculate elemental formulae of the mono-isotopic peak (mass tolerance 5 ppm). The isotopic pattern and Ring Double Bond equivalents (RDB) were considered in formula assignment. Extracted ion chromatograms (XICs) of [M+H+Ca]^3+^ of PLTX and/or its methyl ester and [2M+H+Ca]^3+^ and [3M+3H+Ca]^5+^ for PLTX dimer and trimer, respectively, were obtained and used for calculating the percentage of each ion species using the following formula:

(1) $$\frac{A_{dimer\;or\;trimer}}{A_{monomer}+A_{dimer}+A_{trimer}}\times 100$$

Peak area expressed peak intensity. Measured Limit of Detection (LOD) was 7 ng/mL.

The preliminary evaporation experiment of OA and PLTX (Section 2.1) was performed on API 4000, whereas the study on the stability of PLTX in acids was performed on Orbitrap. All the other reported experiments were performed on API 4000 and repeated on Orbitrap. 

### 4.3. Sample Preparation

Each PLTX sample was obtained from the PLTX stock solution 10 µg/mL by diluting it with the properly described mixture. The relative percentages of the water/methanol mixtures were reported according to the relevant mixture used for the PLTX stock solution dilution. In all explored cases, we considered the amount of methanol present in the initial solution negligible. 

#### 4.3.1. Preliminary Evaporation Experiment on OA and PLTX

PLTX (50 µL) and OA (50 µL) at equimolar concentration were mixed in 80% aqueous MeOH (900 µL) in glass vial, obtaining a mixture of PLTX (125 ng/mL) and OA (25 ng/mL). The mixture was dried down under N_2_-stream at room temperature. The residue, reconstituted in 50% aqueous MeOH (300 µL), was analyzed using instrument 1.

#### 4.3.2. Effect of Solvents on the Intensity of the [M+H+Ca]^3+^ Ion of PLTX

PLTX stock solution was diluted (1:10) in glass vials in different blends: H_2_O 100%; 10, 30, 50 or 80% aqueous MeOH; 80% aqueous EtOH; 80% aqueous IsoPrOH; 100% MeOH; 100% EtOH; 100% IsoPrOH. The solutions were analyzed using LC-HRMS (Instrument 2) 2 h after preparation. 

#### 4.3.3. Effect of Drying Techniques on PLTX Recovery

Aliquots of PLTX in 80% aqueous MeOH (125 ng/mL; 1.0 mL) were evaporated to dryness under N_2_-stream using a Multiple Nitrogen Evaporator (Liebisch Labortechnik, Germany) at room temperature and under vacuum using a Rotational Vacuum Concentrator RVC 2-18 (Martin Christ, Osterode am Harz, Germany) at 30 °C for 5 h. Residues were re-dissolved in 50% aqueous MeOH (300 µL) and analyzed using both instruments. Experiments were performed in triplicate over 1 day and the relative data are expressed as mean ± STD. 

#### 4.3.4. Complete Drying in Different Materials

Different samples of PLTX (125 ng/mL; 1.0 mL) in 80% aqueous MeOH, EtOH and IsoPrOH and in pure MeOH, EtOH and IsoPrOH were prepared using normal and silanized glass vials, PP and Teflon tubes. Three replicates of each solution in each vial/tube were dried-down under N_2_-stream and residues were re-dissolved in 1 mL of the original solvent and analyzed using instrument 1. 

In addition, the same number of aliquots of PLTX dried down in PP tubes were also re-dissolved in 50% aqueous MeOH and analyzed using both LC-MS and LC-HRMS instruments.

Finally, aliquots (1.0 mL) of PLTX in 80% aqueous MeOH at four concentrations (62.5, 125, 250 and 500 ng/mL) were dried down under N_2_-stream at room temperature in Teflon tubes. Residues were re-dissolved either in 300 µL or in 1 mL of 80% aqueous MeOH and analyzed using both instruments. Each experiment was reproduced in triplicate. 

#### 4.3.5. Concentration in Different Materials

Aliquots of PLTX (125 ng/mL; 1.0 mL) in 80% aqueous EtOH, and IsoPrOH and in pure MeOH, were prepared using normal and silanized glass vials, whereas aliquots of PLTX (125 ng/mL; 1 mL) in 100% MeOH, EtOH and IsoPrOH were prepared using PP and Teflon tubes and concentrated to 200 µL under N_2_-stream and analyzed using instrument 1. Each experiment was reproduced in triplicate. 

#### 4.3.6. Study on Stability of PLTX in Acids

Three aliquots of PLTX (1.0 µg/mL; 1.0 mL) in 80% aqueous MeOH (Control) and in 80% aqueous MeOH with 0.2% AA, or 0.1% TFA, or 2% FA were analyzed using LC-HRMS^n^ (*n* = 1, 2) (instrument 2) 3 hours after preparation (day 0) and after 2 weeks (day 14). A second set of PLTX solutions (125 ng/mL; 1.0 mL) in Teflon tubes was dried under N_2_-stream and residues, re-dissolved in 80% aqueous MeOH (1.0 mL), analyzed using both instruments. A third set of PLTX solutions (5 µg/mL; 5.0 mL) in glass tubes was kept at 60 °C for 1 h and analyzed using both instruments.

## Figures and Tables

**Table 1:** Summary of the experiments and conditions used for analyses of PLTX.

	Type of Experiment	PLTX Concentration	Dissolution Solvent	Re-Dissolution Solvent	Volume of Re-dissolution Solvent	Drying Technique	Type of Material	Figures and Tables
1	Preliminary evaporation experiment of OA and PLTX	125 ng/mL PLTX + 25 ng/mL OA Final volume 1 mL	80% MeOH in H_2_O	50% MeOH in H_2_O	300 µL	N_2_-stream	glass	-
2	Effect of solvents on the intensity of the [M+H+Ca]^3+^ ion of PLTX	1.0 µg/mL (dilution 1:10 of the PLTX stock solution)	100% H_2_O; 10, 30, 50 or 80% MeOH in H_2_O; 80% EtOH in H_2_O; 80% isoPrOH in H_2_O; 100% MeOH or EtOH or IsoPrOH	none	none	none	glass	Figures 1 and 2, Tables S1 and S2
3	Effect of complete drying techniques on PLTX recovery	125 ng/mL (1 mL)	80% MeOH in H_2_O	50% MeOH in H_2_O	300 µL	N_2_-stream	glass	Figures 3 and 4
			80% MeOH in H_2_O	50% MeOH in H_2_O		Centrifugal Vacuum Concentrator		
4	Effects of complete drying on PLTX recovery	125 ng/mL (1 mL)	80% MeOH in H_2_O;	80% MeOH in H_2_O;	1.0 mL	N_2_-stream	glass, silanized glass; PP; Teflon	Figure 5a,b and Table S3
			80% EtOH in H_2_O;	80% EtOH in H_2_O;				
			80% isoPrOH in H_2_O;	80% isoPrOH in H_2_O;				
			100% MeOH;	100% MeOH;				
			100% EtOH;	100% EtOH;				
			100% IsoPrOH	100% IsoPrOH				
	**In-depth analysis-1**. Complete drying in PP tubes changing the condition of re-dissolution	125 ng/mL (1 mL)	80% MeOH in H_2_O	50% MeOH in H_2_O	1.0 mL	N_2_-stream	PP tube	Table 2
			80% EtOH in H_2_O;					
			80% isoPrOH in H_2_O;					
	**In-depth analysis-2**. Complete drying in Teflon at different PLTX concentration	62.5 ng/mL (1 mL)	80% MeOH in H_2_O	80% MeOH in H_2_O	300 µL or 1.0 mL	N_2_-stream	Teflon	Figure 6 and Table S4
		125 ng/mL (1 mL)						
		250 ng/mL (1 mL)						
		500 ng/mL (1 mL)						
5	Concentration to 200 µL in different materials	125 ng/mL (1 mL)	80% EtOH in H_2_O;	none	none	N_2_-stream	glass, silanized glass;	Figure 7a,b and Table S5
			80% isoPrOH in H_2_O					
			100% MeOH					
			100% MeOH or EtOH or IsoPrOH;	none	none		PP; Teflon	

**Table 2:** Complete drying performed with N_2_-stream of PLTX (125 ng/mL, 1.0 mL) in polypropylene (PP) tubes in different solvents/blends. Samples are re-dissolved in the same initial solvent/blend or in 50% aqueous MeOH. Recoveries (% ± RSD) are calculated as mean of three different replicates. * not detected.

PLTX in	Complete Drying in PP and Re-Dissolution in	
	Original Solvent	50% Aqueous MeOH
80% MeOH in H_2_O	40 ± 13	56 ± 11
80% EtOH in H_2_O	59 ± 8	61 ± 4
80% IsoPrOH in H_2_O	23 ± 2	60 ± 6
MeOH 100%	27 ± 1	42 ± 8
EtOH 100%	16 ± 7	46 ± 9
IsoPrOH 100%	nd *	40 ± 13

**Table 3:** Percentage of PLTX and its derivative at day 0 and 14 in all acidified samples versus control.

	80% Aqueous MeOH		80% Aqueous MeOH with					
	Control		0.2% AA		2% FA		0.1% TFA	
	PLTX%	PLTX Derivative%	PLTX%	PLTX Derivative%	PLTX%	PLTX Derivative%	PLTX%	PLTX Derivative%
Day 0	100	0	100	0	89	11	84	16
Day 14	100	0	94	6	61	39	37	63

**Table 4:** Structure of palytoxin and its methyl ester at C1 and assignment of fragments contained in HR CID MS^2^ spectrum of the latter. Elemental formulae (*m/z*) are reported with charge state (1+, 2+ or 3+) and ring double bond equivalent (RDB). Errors were all below 5 ppm. LC HRMS/MS of the PLTX methyl ester tentative mechanism for methyl ester formation.

	Palytoxin Methyl Ester			
	Clv	A Side	B Side	
		*m/z* (1+,2+,+3) Formula RDB	*m/z* (1+,2+,+3) Formula RDB	
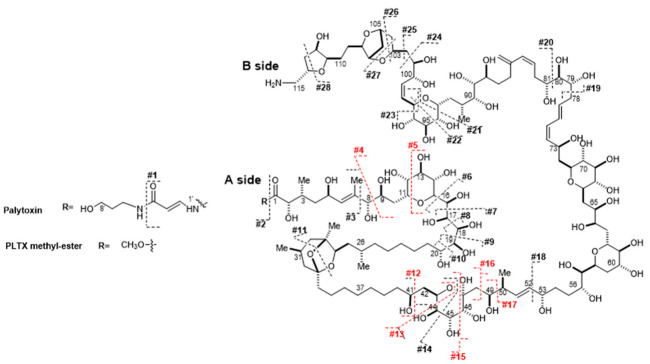 **Tentative Mechanism of formation of the PLTX methyl ester**	**#4**		1178.1179 (2+) C_113_H_193_O_47_NCa 17.0, -1.664	
	**#5**		791.7502 (3+) C_113_H_196_O_48_NCa 16.5, -1.274	
	**#12**	488.2636 (2+)C_47_H_84_O_18_Ca 6.0	-	
	**#13**	510.2767 (2+) C_49_H_88_O_19_Ca6.0	-	
	**#15**	540.2873 (2+) C_51_H_92_O_21_Ca 6.0	782.8752 (2+) C_73_H_123_O_32_NCa 13.0	1526.8181 (1+) C_73_H_124_O_32_N 12.5
	**#16**	568.3004 (2+) (-1H_2_O) C_54_H_96_O_22_Ca 7.0	745.8638 (2+) C_70_H_117_O_30_NCa 13.0	1452.7733 (1+) C_70_H_118_O_30_N 12.5
	**#17**	647.3343 (2+) C_60_H_106_O_25_N_2_Ca 9.0	1388.7573 (1+) (-1H_2_O) C_69_H_114_O_27_N 13.5	
	**#4+#12**	372.1981 (2+) C_36_H_64_O_13_Ca 5.0		
	**#4+#15**	424.2218 (2+) C_40_H_72_O_16_Ca 5.0		
	**#4+#16**	452.2349 (2+) (-1H_2_O) C_43_H_76_O_17_Ca 6.0		
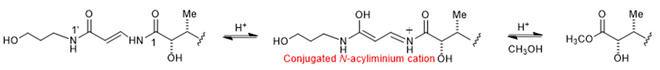				

## Data Availability

Not applicable.

## Supplementary Materials

The following are available online at https://www.mdpi.com/article/10.3390/toxins13090650/s1, Figure S1: Planar structure of assorted palytoxins, Figure S2: HRMS spectrum of PLTX in 100% water, Figure S3, HRMS^2^ spectrum of PLTX methyl ester, Table S1: MS parameters and transitions used in Multiple Reaction Monitoring (MRM) experiments, Table S2: The intensity of PLTX mass peaks diluted in different solvents and blends, Table S3: Recoveries of PLTX following complete drying in PP and Teflon tubes, normal and silanized glass, Table S4: Recoveries of PLTX at different initial concentration following complete drying in Teflon tubes, Table S5: Recoveries of PLTX following concentration in PP and Teflon tubes, normal and silanized glass vials.
