# Supplementary material for: Toward Isolation of Palytoxins: Liquid Chromatography Coupled to Low- or High-Resolution Mass Spectrometry for the Study on the Impact of Drying Techniques, Solvents and Materials
Source: Toxins (Basel). 2021 Sep 14;13(9):650. doi: 10.3390/toxins13090650 (PMC8471787; doi:10.3390/toxins13090650)
Supplement: Supplementary file 1 [file toxins-13-00650-s001.zip › toxins-1342275-supplementary.pdf]

Supplementary Materials: Toward Isolation of Palytoxins: Liquid Chromatography Coupled to Low- or High-Resolution Mass Spectrometry for the Study on the Impact of Drying Techniques, Solvents, And Materials

Antonia Mazzeo, Michela Varra, Luciana Tartaglione, Patrizia Ciminiello, Zita Zendong, Philipp Hess and Carmela Dell'Aversano

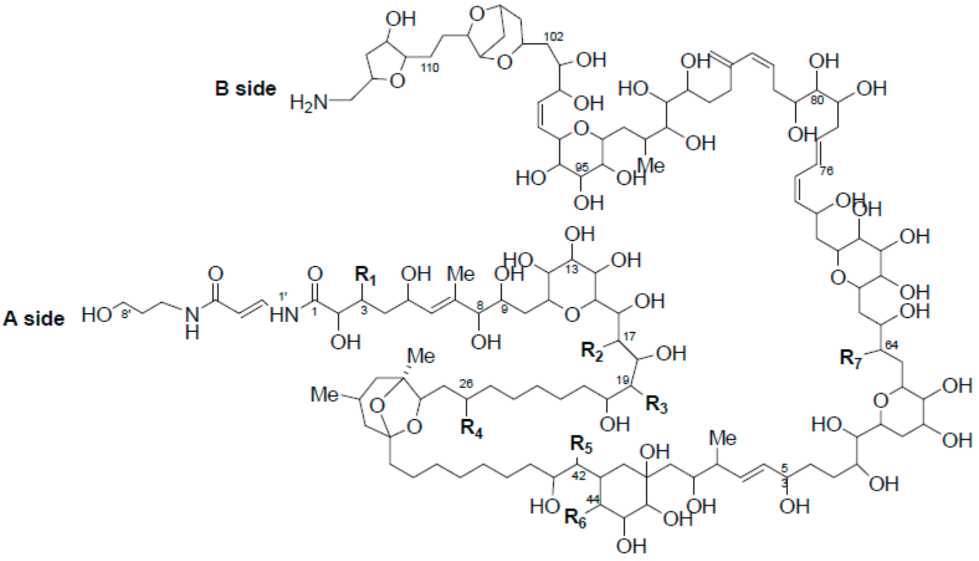

	R_1	R_2	R_3	R_4	R_5	R_6	R_7
Palytoxin	Me	OH	OH	Me	H	OH	OH
42-Hydroxypalytoxin	Me	OH	OH	Me	OH	OH	OH
Ovatoxin-a	Me	H	OH	Me	OH	H	H
Ostreocin-D	H	OH	H	H	OH	H	OH

Figure S1. Planar structure of some palytoxin congeners including palytoxin and 42-hydroxypalytoxin from *Palythoa* spp. and ovatoxin-a and ostreocin-D from *Ostreopsis* spp.

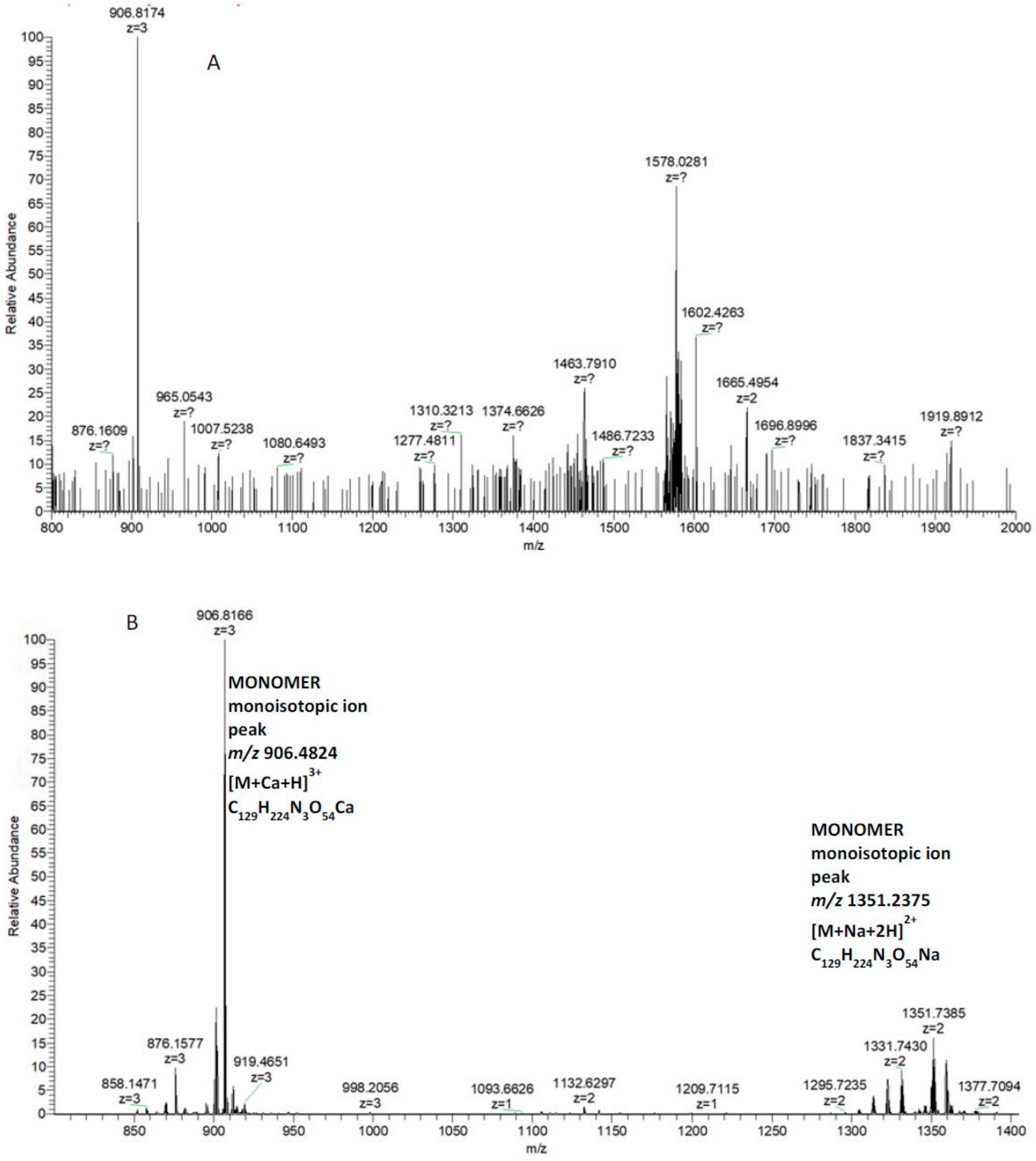
A
906.8174 z=3
876.1609 z=?
965.0543 z=?
1007.5238 z=?
1080.6493 z=?
1277.4811 z=?
1310.3213 z=?
1374.6626 z=?
1463.7910 z=?
1486.7233 z=?
1578.0281 z=?
1602.4263 z=?
1665.4954 z=2
1696.8996 z=?
1837.3415 z=?
1919.8912 z=?
Relative Abundance
m/z
B
906.8166 z=3
MONOMER monoisotopic ion peak
m/z 906.4824
[M+Ca+H]3+
C129H224N3O54Ca
MONOMER monoisotopic ion peak
m/z 1351.2375
[M+Na+2H]2+
C129H224N3O54Na
858.1471 z=3
876.1577 z=3
919.4651 z=3
998.2056 z=3
1093.6626 z=1
1132.6297 z=2
1209.7115 z=1
1295.7235 z=2
1331.7430 z=2
1351.7385 z=2
1377.7094 z=2
Relative Abundance
m/z

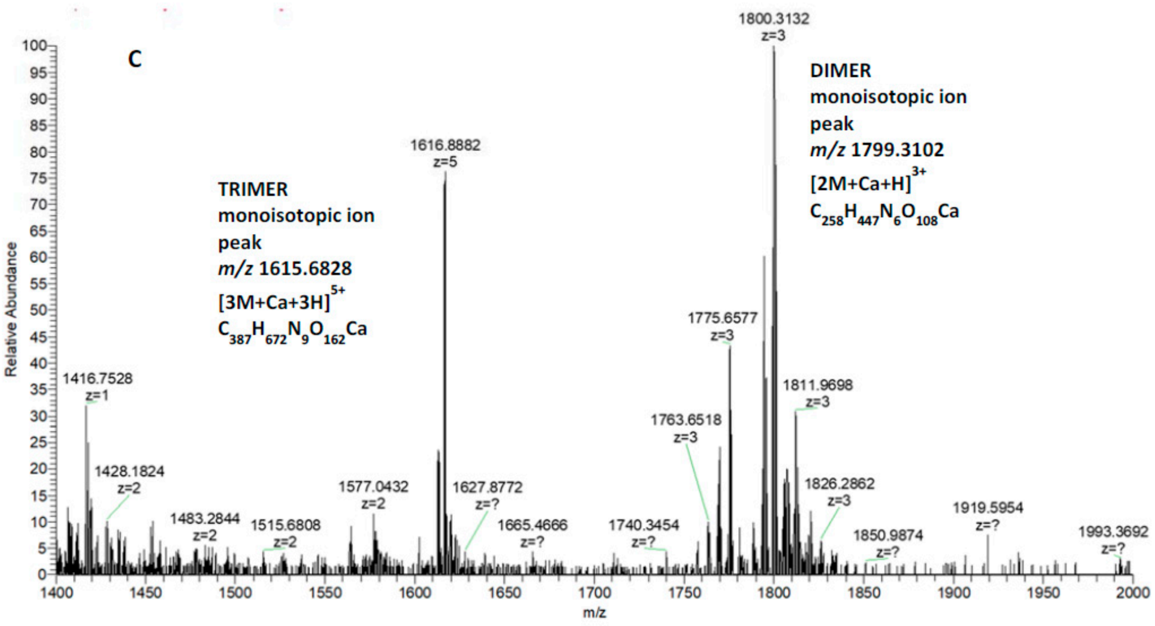

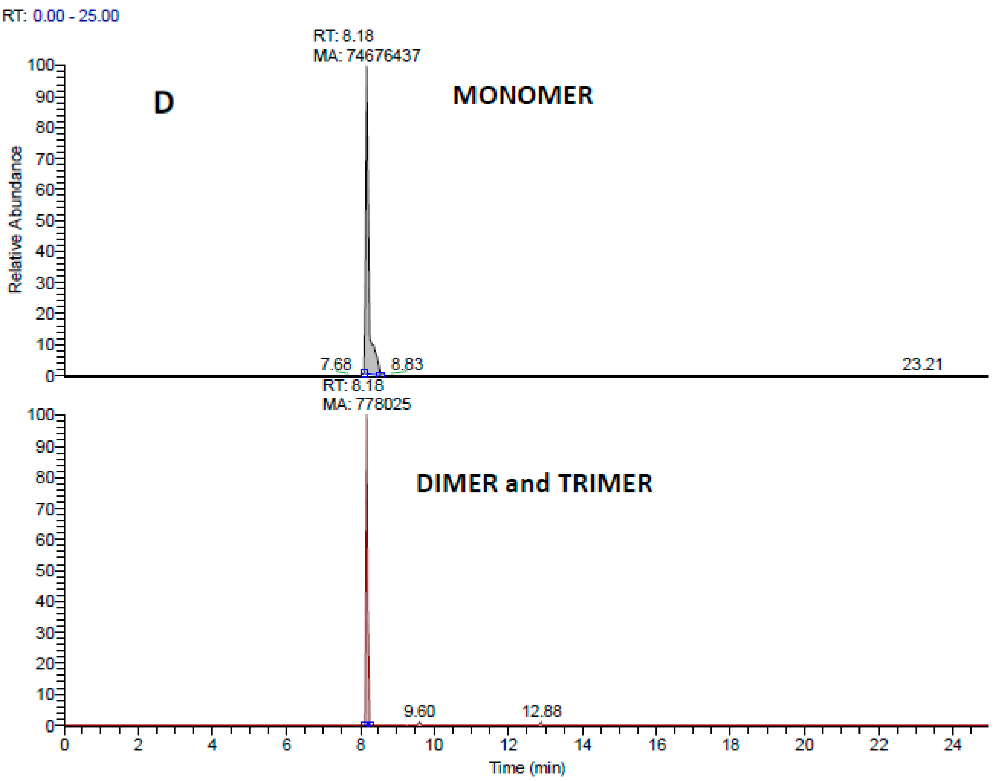

Figure S2. (**A**) Full HRMS spectrum of PLTX in 100% water in the mass range *m*/*z* 800–2000. (**B**) Full HRMS spectrum of PLTX in the mass range m/z 800-1400. (**C**) Full HRMS spectrum of PLTX in the mass range *m*/*z* 1400–2000. The spectra were acquired simultaneously in three different scan events. (**D**) extracted ion chromatogram of the main monomer ions (*m*/*z* 906.8166; 1351.7385) and of the main dimer and trimer ions (m/z 1616.8882; 1800.3132).

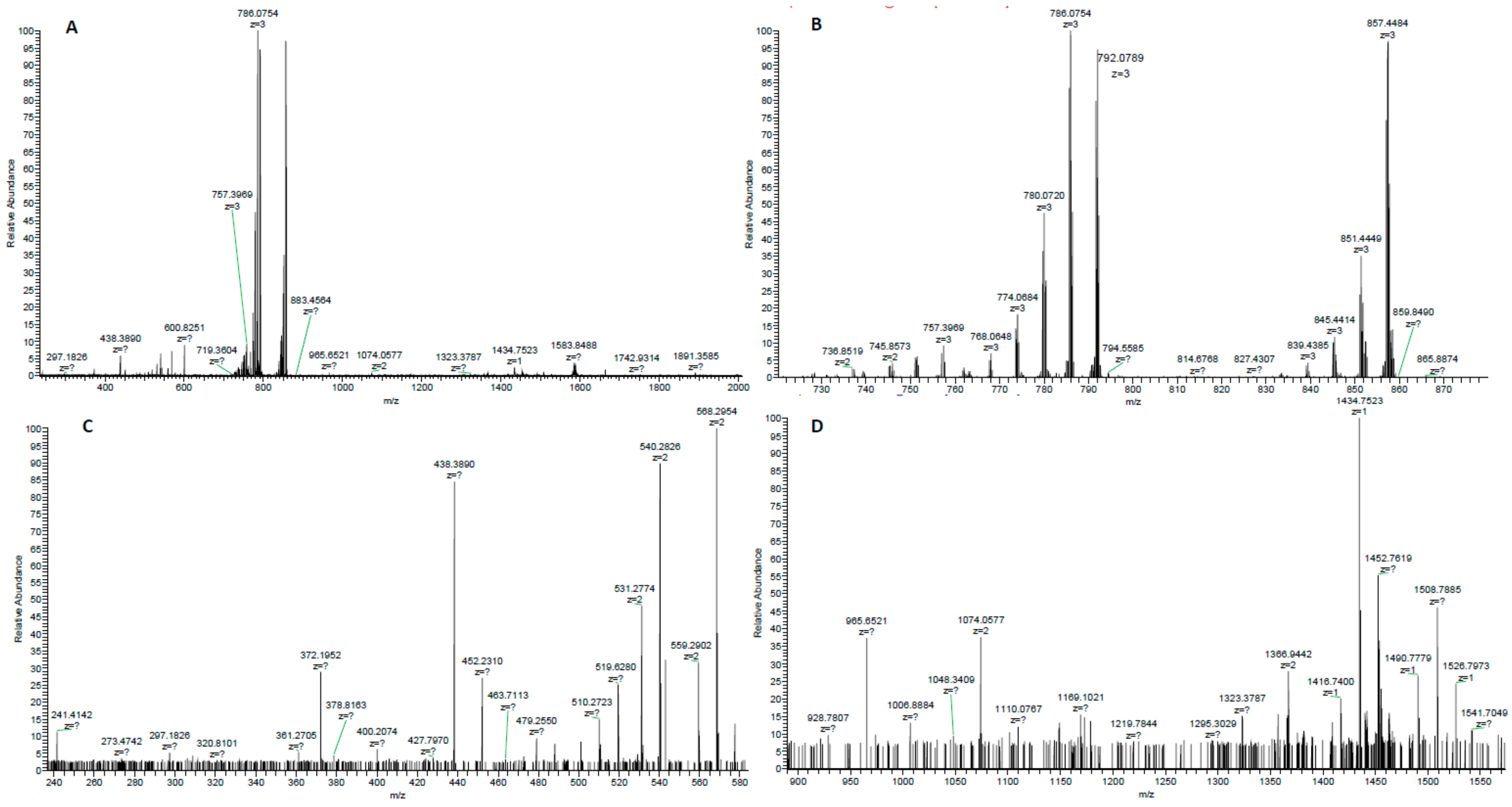

Figure S3. (**A**) HRMS$_2$ spectrum of PLTX methyl ester in the *m/z* range 0–2000. (**B**) HRMS$_2$ spectrum of PLTX methyl ester zoomed in the *m/z* range 720–880. (**C**) HRMS$_2$ spectrum of PLTX methylester zoomed in the *m/z* range 230-580. (**D**) MS$_2$ spectrum of PLTX methyl ester zoomed in the *m/z* range 900-1580 Several fragment ions due to water losses the precursor ion at *m/z* 869.4 are the following: *m/z* 857.1187 (-2H_2O) $C_{124}H_{212}NO_{51}Ca$, *m/z* 851.1155 (-3H_2O) $C_{124}H_{210}NO_{50}Ca$, *m/z* 845.1123 (-4H_2O) $C_{124}H_{208}NO_{49}Ca$, *m/z* 839.1069 (-5H_2O) $C_{124}H_{206}NO_{48}C$ not shown in Table 4 in the main text.

Table S1. Transitions and MS parameters used in Multiple Reaction Monitoring (MRM) experiments on Instrument 1 (Source settings: curtain gas 30 psi, ion spray 5000 V, turbogas temperature 300 °C, declustering potential 56 V, Dwell time 500 or 250 ms for PLTX or OA respectively, entrance potential 10 V, Gas 1 and Gas 2 30 and 40 psi, respectively.

	Q1		Q3		Collision Energy (eV)	Cells Exit Potential (V)
	**Precursor**	m/z	**Precursor**	m/z		
PLTX	[M+2H]2+	1340.9	[A moiety+H-H2O]+[a]	327.3	47	20
	[M+2H-H2O]3+	1331.9	[A moiety+H-H2O]+	327.3	47	20
	[M+3H-H2O]3+	887.8	[A moiety+H-H2O]+a	327.3	31	18
OA	[M+NH4]+	822.5	[M+H-H2O]+	787.3	17	20
	[M+NH4]+	822.5	[M+H-2H2O]+	769.4	23	12
	[M+NH4]+	822.5	[M+H-3H2O]+	751.4	29	18
	[M+NH4]+	822.5	[M+H]+	805.4	13	12

[a]A moiety is the PLTX part structure stretching from A-side terminal to C8 (Figure S1).

Table S2. The intensity of PLTX (1.0 µg/mL) mass peaks diluted in different solvents and blends. The samples were prepared in glass vials.

PLTX diluted in	Average area	STD
H2O 100%	273509	193193
MeOH 10%	3574802	517672
MeOH 30%	3534432	600363
MeOH 50%	3685748	463429
MeOH 80%	2886110	237643
EtOH 80%	2751877	455188
IsoPrOH 80%	2384957	490035
MeOH 100%	1362005	282464
EtOH 100%	840114	363923
IsoPrOH 100%	314914	233285

Table S3. Recoveries of PLTX (125 ng/mL, 1.0 mL) following complete drying performed with N2 stream in PP and Teflon tubes, normal and silanized glass vials. The samples were re-dissolved in 300 µL of the initial solvent/blend. Recoveries are expressed in % ±RSD.

PLTX	Recovery % (n = 3)* Complete drying and re-dissolution in original solvent			
	**PP**	**Teflon**	**Normal Glass**	**Silanized Glass**
80% MeOH in H2O	40±13	63±11	58±6	55±6
80% EtOH in H2O	59±8	97±11	32±2	13±3
80% IsoPrOH in H2O	23±2	87±21	30±3	11±1
100% MeOH	27±1	76±3	28±8	33±2
100% EtOH	16±7	37±12	nd	nd
100 IsoPrOH	nd	nd	nd	nd

* n = number of replicates, nd = not detected.

Table S4. Recoveries of PLTX at different initial concentration (initial volume 1.0 mL) following complete drying performed with N2-stream in Teflon tubes. The samples were dissolved in 80% aqueous MeOH and re-dissolved in 300 µL or 1.0 mL of 50% aqueous MeOH. Recoveries are expressed in % ±RSD.

Re-dissolution in MeOH 50% aqueous methanol				
	Recovery% (n = 3)		Loss% (ng)**	
**PLTX ng/mL**	**300 μL**	**1 mL**	**300 μL**	**1 mL**
62.5	47 ± 10	52 ± 16	83 (33.1)	48 (30)
125	48 ± 3	59 ± 8	52 (65)	41 (51.2)
250	72 ± 7	77 ± 12	28 (70)	23 (57.5)
500	86 ± 6	86 ± 4	(14 (70)	14 (70)

* n = number of replicates, ** = The PLTX loss is expressed both in percentage and in the corresponding ng, the latter value is reported in the brackets.

Table S5. Recoveries of PLTX (125 ng/mL, 1.0 mL) following concentration at 200 μL under N2-stream in PP and Teflon tubes, normal and silanized glass vials. Recoveries are expressed in % ±RSD.

PLTX in	Recovery % (n = 3)* Concentration in			
	**PP**	**Teflon**	**Normal Glass**	**Silanized Glass**
80% MeOH in H2O	-	-	-	
80% EtOH in H2O	-	-	66 ± 17	69 ± 12
80% IsoPrOH in H2O	-	-	80 ± 2	76 ± 4
100% MeOH	86 ± 2	89 ± 5	60 ± 10	47 ± 17
100% EtOH	83 ± 6	86 ± 1	-	-
100 IsoPrOH	47 ± 17	57 ± 12	-	-

*n=number of replicates.
